# Challenges in Endobronchial Tuberculosis: From Diagnosis to Management

**DOI:** 10.1155/2014/594806

**Published:** 2014-08-14

**Authors:** Surender Kashyap, Anjali Solanki

**Affiliations:** ^1^Kalpana Chawla Government Medical College, Karnal, Haryana 132001, India; ^2^Department of Pathology, Kalpana Chawla Government Medical College, Karnal, Haryana 132001, India

## Abstract

Despite the rapid advancement in diagnostic and therapeutic modalities, endobronchial tuberculosis (EBTB), defined as tuberculous infection of the tracheobronchial tree, continues to remain challenging for clinicians. Nonspecific respiratory symptoms along with normal chest radiograph in 10–20% of cases may be alleged for the diagnostic delay. Variable diagnostic yield with sputum microscopy might further compound the problem. In such cases, high resolution computed tomography (HRCT) works as a more sensitive tool and demonstrates involvement of tracheobronchial tree described classically as “tree-in-bud” appearance. Bronchoscopic biopsy is considered the most reliable method for confirmation of the diagnosis with 30% to 84% positivity in different series. Evolution of the disease is also unpredictable with frequent progression to bronchostenosis, therefore requiring regular follow-up and early intervention to halt the natural course. This review article elaborates various aspects of the disease with specific focus on diagnostic dilemma and recent advances in interventional bronchoscopy. In addition, this discussion evokes optimism for further research and introduction of innovative therapeutic modalities.

## 1. Introduction

“The struggle has caught hold along the whole line and enthusiasm for the lofty aim runs so high that a slackening is no longer to be feared. If the work goes on in this powerful way, then the victory must be won.” While these concluding words of Nobel Laureate addressed on December 12, 1905, reflect optimistic attitude and confidence of Robert Koch, discoverer of* Mycobacterium tuberculosis*, unfortunately the situation has not improved even after more than 100 years. In 2011, approximately 9 million people suffered from tuberculosis and 1.4 million died, with 60% of cases in Asia and 24% in Africa [1]. With this huge overall burden of the disease and associated morbidity and mortality, it is worthwhile to discuss a specific form of tuberculosis known as endobronchial tuberculosis (EBTB) because of challenges associated with diagnosis as well as prognosis.

Endobronchial tuberculosis is defined as tuberculous infection of the tracheobronchial tree with microbial and histopathological evidence [2]. Clinicians encounter difficulties in each and every aspect related to this entity and diagnosis is usually delayed because of nonspecific symptoms. Even after accurate diagnosis is established, clinical course is quite variable with frequent progression to bronchostenosis. The incidence rate of bronchostenosis may be up to 68% in initial 4 to 6 months of the disease and, in long term, more than 90% of the patients are usually affected [3, 4]. Advancement in interventional bronchoscopy has definitely changed the overall scenario with excellent management of tracheobronchial stenosis and surgery should only be used as a last resort when other modalities fail.

## 2. Predilection for Young Females 

Exact incidence of EBTB is difficult to predict as there are variable data related to incidence of the disease which might be due to retrospective method used in most of the series. According to various studies, EBTB was present in 10–38.8% of patients with active pulmonary tuberculosis [3]. However, few authors have reported strikingly low incidence of 5.88% possibly because bronchoscopy was not routinely performed in all cases of pulmonary tuberculosis [5, 6].

EBTB has predilection for young women [3, 5, 6] which is usually explained by easy implantation of organisms from infected sputum, since women do not generally expectorate sputum possibly because of their sociocultural factors. However, satisfactory answer for this phenomenon is still not available.

Majority of patients are usually in second or third decade [3, 7]. In addition, there is a second peak in old age also as described by van den Brande et al. [8].

## 3. Possible Mechanisms of Pathogenesis

The exact pathogenesis of endobronchial tuberculosis is not understood yet; however, suggested possible mechanisms of infection include (a) direct extension from an adjacent parenchymal focus, (b) implantation of organisms from the infected sputum, (c) haematogenous dissemination, (d) lymph node erosion into a bronchus, and (e) spread of infection via the lymphatics [9]. Lymph node erosion into adjacent bronchus is particularly important mechanism in paediatric patients due to the small calibre of bronchus and the weak wall of the bronchus [10].

The most common site of involvement is right upper lobe and right main bronchus [3]. Initially bronchial lesion presents with infiltration of lymphocytes into mucosa followed by considerable congestion and edema of mucosal surface [11]. Development of caseous necrosis with formation of tuberculous granuloma can be found at the mucosal surface. Fibrotic change of the lamina propria as well as healing of mucosal ulceration eventually progresses to bronchostenosis [12].

In addition to local factors, various cytokines may also play an important role in pathogenesis. It has been shown that elevated interferon gamma and TGF-beta levels in bronchial lavage fluids may be related to pathogenesis and progression of EBTB. Lowered initial serum TGF-beta levels and changes in the levels of TGF-beta observed in the serum after treatment have been implicated in the development of bronchial stenosis during the course of the disease [13]. It is evident that complex interplay of multiple mechanisms is responsible for the development and progression of EBTB in adults.

## 4. Heterogeneity in Clinical Presentation 

The onset of EBTB may be acute, insidious, or delayed. The duration of symptoms also varies strikingly. Clinical features are variable and dependent upon the site, extent of involvement, and stage of the disease. Systemic symptoms like anorexia, weight loss, and night sweats might not be prominent in EBTB [2, 14]. Fever, if present, is usually of low grade at the onset but may become marked with the progression of the disease [14]. The respiratory symptoms in EBTB are usually nonspecific and confusing. A barking cough unresponsive to common antitussive medication is the most common presentation which slowly progresses over weeks or months [3]. Sputum production is rare but bronchorrhea has been reported in active cavitary endobronchial tuberculosis [15]. Hemoptysis also occurs occasionally but is rarely massive. With lymph node rupture, chest pain may occur in the sternal or parasternal region, which is sharp or dull and is encountered in about 15% per cent of patients. Dyspnoea is often associated with atelectasis. Wheeze and stridor may be the presenting features of bronchostenosis.

Clinical examination reveals decreased breath sounds, wheezing, and rhonchi [7]. Since these symptoms and signs are nonspecific and mimic various pulmonary diseases like bronchial asthma [16], pneumonia [17], foreign body aspiration [18], and malignancy [19], it is very difficult to diagnose the disease with clinical presentation alone and thereby frequently missed. Awareness of the entity and consideration of EBTB as differential diagnosis in confusing cases might be helpful for clinicians in planning further strategy for definitive diagnosis.

## 5. Efforts to Solve Diagnostic Dilemma

In cases of suspected endobronchial tuberculosis, although diagnostic workup should commence with demonstration of acid fast bacilli in sputum, bronchoscopy and computed tomography are essential for accurate diagnosis of bronchial involvement as well as assessment for surgical intervention. Fibreoptic bronchoscopy is indicated in patients in whom chest radiographs, physical signs, or symptoms suggest the possibility of endobronchial tuberculosis.

### 5.1. Sputum Examination: Variable Diagnostic Yield

Bacteriologic confirmation should be the first step for confirmation of diagnosis of EBTB. Freshly expectorated sputum is considered the best sample for microscopy. However, the diagnostic yield of sputum examination is not up to the level of expectation in EBTB. The basis of this finding is not clear, but it can be attributed to difficulty in expectoration of sputum and lack of mucosal ulceration in most of the cases [3]. Demonstration of acid fast bacilli has been reported between 16 and 53% in various studies despite an accurate sputum examination [20, 21]. In one of the recent studies, including 23 biopsy proven cases, all patients were sputum smear negative. Authors have explained this low yield because of mucus entrapment by proximal bronchial granulation tissue and further suggested that a negative sputum smear does not preclude the diagnosis of EBTB [22]. In view of low positivity rates by microscopy alone, nuclear amplification tests like polymerase chain reaction (PCR) or other methods for amplifying DNA and RNA in a reference laboratory are recommended in suspected cases [23].

### 5.2. Role of Tuberculin Skin Test (TST) and Interferon Gamma Release Assays (IGRA)

In view of low positivity rate of acid fast bacilli detection by sputum microscopy in EBTB, other diagnostic methods may be beneficial to reach a diagnosis of tuberculosis in confusing scenario.

Tuberculin skin test (TST) is one of the oldest diagnostic tests and demonstrates delayed type hypersensitivity reaction to purified protein derivative (PPD) in exposed individuals. However, this test suffers from shortcomings of low sensitivity particularly in immunocompromised individuals, interobserver variability, and lack of specificity due to cross-reactivity with the nontuberculous mycobacteria (NTM) and Bacillus Calmette Guérin (BCG) strain [24].

More specific and recent additions to this list are interferon gamma release assays (IGRA). These are the new T-cell based assays that measure the interferon gamma release, by sensitized lymphocytes in response to specific* M. tuberculosis* antigens such as the early secreted antigenic target-6 (ESAT-6) and culture filtrate protein 10 (CFP10) [25]. In general, these tests are more specific and potentially more sensitive particularly in immunosuppressed individuals than the traditional TST. Furthermore, IGRA requires single visit and lacks dependency of results on observer and technique and prior BCG vaccination. Sensitivity of IGRA in active TB ranges from 64 to 92% which further varies between high and low burden countries. Chances of potentially missing 10 to 30% of active cases limit the utility of IGRA as “rule-in” tests. In view of issues of affordability, prerequisite of sophisticated instruments, and inability to differentiate latent infection from active disease, prospective studies are required in high-burden settings to determine its usefulness for diagnosis of tuberculosis [24].

### 5.3. Pulmonary Function Testing: Restrictive Pattern Is Dominant

Utility of pulmonary function tests lies in the patients presenting with cough, dyspnoea, and wheezing thereby being confused with bronchial asthma. The dominant pattern in EBTB is usually restrictive (47%), followed by normal pulmonary function, mixed pattern, and obstructive pattern [6]. The predominance of a restrictive pattern might be due to the complete obstruction of the bronchial tree or due to the chronic inflammatory changes and bronchiectatic changes of the parenchyma beyond the stenosis [7].

### 5.4. Computerized Tomography: Improved Sensitivity

**Figure 1:**
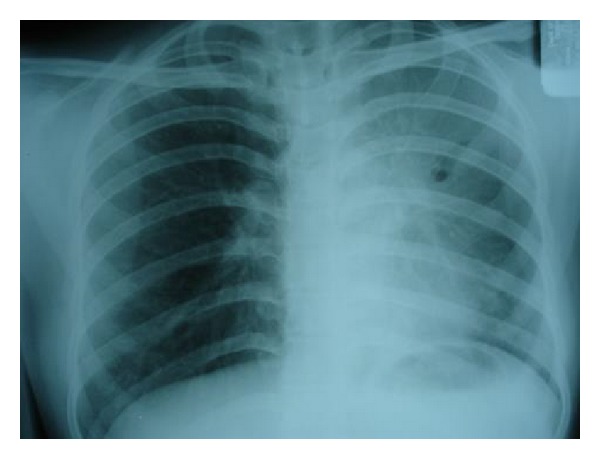
Chest radiograph of a young female showing collapse consolidation of left upper lobe caused by EBTB.

Even normal roentgenograms do not exclude endobronchial involvement in tuberculosis. In a retrospective study by Lee and Chung 10% of the patients diagnosed to have endobronchial tuberculosis demonstrated no abnormality on chest films [6]. Predominant radiological finding is patchy parenchymal infiltration involving both upper and lower lung fields. If bronchostenosis develops, persistent segmental or lobar collapse (Figure 1), lobar hyperinflation, obstructive pneumonia, and mucoid impaction may be noted [26]. As these findings are frequently misleading and nondiagnostic, computerized tomography and bronchoscopy are essential to establish the diagnosis.

**Figure 2:**
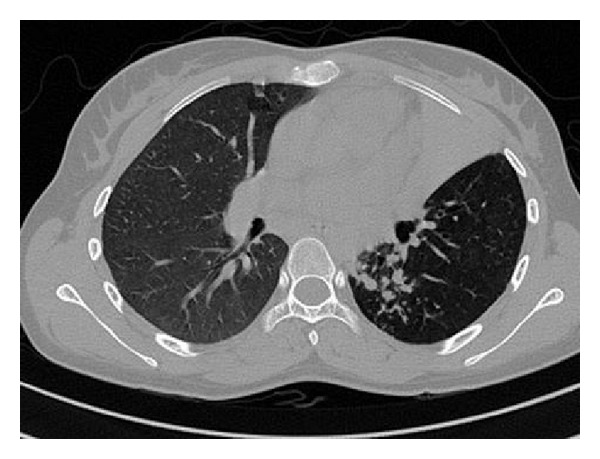
CT thorax showing left upper lobe collapse and infiltrations in the lower lobe due to EBTB.

High resolution computed tomography (HRCT) is more sensitive than conventional chest radiograph and axial computed tomography (CT) in demonstrating early endobronchial spread. As volumetric computed tomography provides both multiplanar and three-dimensional (3D) images, it is useful for global understanding of the status of the tracheobronchial tree, particularly for evaluation of focal stenosis of the airways, providing information for preparing the road map for bronchoscopy, for treatment planning, and for assessing treatment efficacy [27]. Studies using HRCT have shown a much higher prevalence of the disease because endobronchial involvement can be detected accurately and in early stage. Endobronchial involvement in pulmonary tuberculosis was reported as high as 95% and 97% with HRCT scanning in different studies [28, 29]. Im et al. [28] reported that earliest finding of bronchogenic dissemination is centrilobular nodules measuring 2–4 mm in diameter containing caseous material within or around terminal bronchiole. With extensive involvement, branching linear structures of similar calibre arising from a stalk giving “tree-in-bud” appearance are noted. Stalk might be representing a lesion of last order bronchus and terminal tufts representing terminal bronchioles and alveolar ducts. In addition, nodules with poorly defined margins, lobular areas of consolidation, thickening of bronchial wall, and interlobular septa also depict bronchogenic spread in some cases (Figure 2) [29].

### 5.5. Bronchoscopy: Valuable for Diagnosis as well as Prognosis

**Figure 3:**
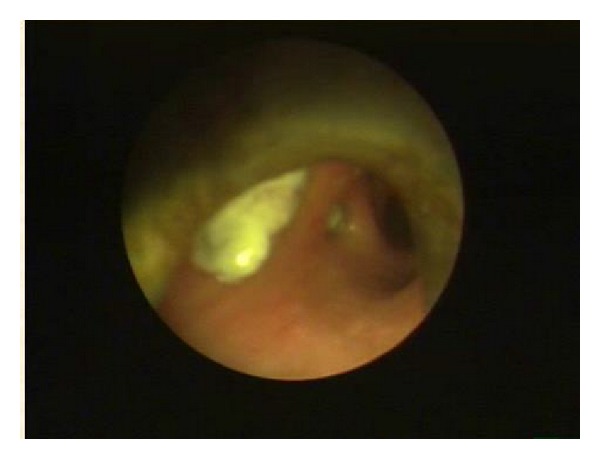
Bronchoscopic appearance of tumorous EBTB covered with whitish necrotic tissue, mimicking a malignant mass.

Bronchoscopy is mandatory for accurate diagnosis. On the basis of bronchoscopic findings EBTB is usually classified into seven subtypes with specific appearance: (i)* actively caseating*—swollen hyperemic bronchial mucosa covered with whitish cheese-like material, (ii)* edematous-hyperemic*—extensive mucosal swelling with surrounding hyperemia, (iii)* fibrostenotic*—marked narrowing of the bronchial lumen with fibrosis, (iv)* tumorous*—endobronchial mass with surface covered by caseous material and nearly totally occluding the bronchial lumen (Figure 3), (v)* granular*—appearance like scattered grains of boiled rice, (vi)* ulcerative*—ulcerated bronchial mucosa, and (vii)* nonspecific bronchitis*—only mild mucosal swelling and/or hyperemia [30].

Out of these subtypes, the actively caseating type (43.0%) is reported as the most common form and the ulcerative type (2.7%) as the least common with other subtypes falling in between [5]. This classification of EBTB is closely related to the extent of disease progression.

**Figure 4:**
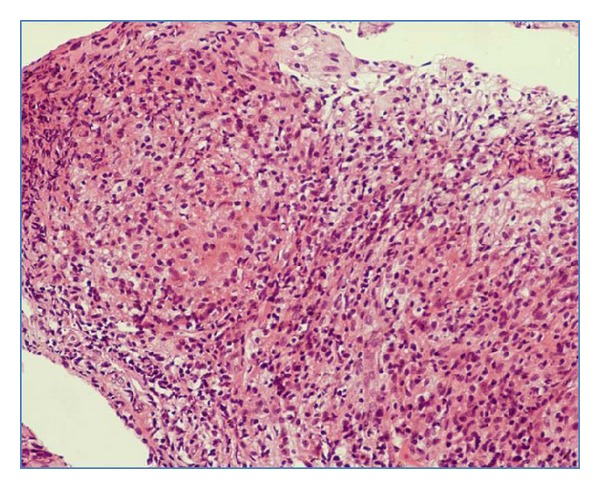
Histopathological examination demonstrating caseating epithelioid granulomas with lymphocytic cuffing suggestive of tuberculosis, hematoxylin-eosin (H.E.) stain ×40.

Though, with aid of bronchoscopy, different samples including biopsy, brushing, and washings could be obtained, bronchoscopic biopsy is the most reliable method for confirming the diagnosis (Figure 4). Bronchial biopsies may be positive in 30% to 84% of patients [21, 31]. In a study by Altin et al. [31], the positivity rate was much higher with bronchoscopic biopsy as compared to bronchial fine needle aspiration (84% versus 16%). The highest positivity for acid fast bacilli (AFB) as well as mycobacterial culture in bronchial lavage fluid has been reported in the granular type of EBTB (75%) and the least in fibrostenotic stage indicating significance of microbiologic methods restricted for early lesions. Thus, histopathology has a crucial role, particularly in fibrostenotic stage for diagnostic purpose [22].

## 6. Unpredictable Clinical Course

Chung and Lee [5] prospectively analyzed the evolution of disease with serial bronchoscopies, starting from the diagnosis till the completion of antituberculous therapy. According to their findings, the initial nonspecific bronchitic form is followed by submucosal tubercle formation giving rise to the appearance of granular and edematous-hyperemic type. At this point, the development of caseous necrosis with the formation of tuberculous granuloma can be found at the mucosal surface. Further, when the inflammation erupts through mucosa, an ulcer covered by caseous material develops. Finally, the bronchial mucosal ulcer evolves into hyperplastic inflammatory polyps, and the endobronchial tuberculous lesion heals by fibrostenosis. Tumorous EBTB can also develop by erosion of an intrathoracic tuberculous lymph node into the bronchus.

The prognosis of actively caseating type and edematous-hyperemic type EBTB is worst, resulting in fibrostenosis in two-thirds of patients. The prognosis is relatively better for granular, ulcerative, and nonspecific bronchitic type EBTB. However, the clinical course of tumorous type is complicated with diverse progress and unexpected changes, frequently resulting in bronchial stenosis despite adequate treatment.

Furthermore, this progression is not directed in one dimension. All subtypes of EBTB are situated between the extreme ends of healing and bronchostenosis and can transform into other subtypes during treatment. But there is a critical point between these two ends, which is mainly determined by the extent of disease progression and closely related to formation of granulation tissue [32]. Bronchial stenosis is inevitable, if the disease progresses beyond this critical point. The eventual therapeutic outcome can be predicted by follow-up bronchoscopy during the initial 2 to 3 months of treatment for all the subtypes except the tumorous variety in which evolution is complicated and bronchial stenosis may develop later [5].

## 7. Frequent Progression to Complications

Bronchial stenosis and stricture are the most common complications and may develop in 60 to 95% cases despite adequate antituberculous therapy. If stenosis involves trachea, airway obstruction can develop. Another common complication is bronchiectasis which frequently develops as paracicatricial process, secondary to pulmonary destruction and fibrosis (traction bronchiectasis). Central bronchostenosis with distal bronchial dilatation can also lead to development of bronchiectasis. Bronchiectasis is typically asymptomatic and usually involves the upper lobes. Hemoptysis is the most common presentation in symptomatic cases [20].

## 8. Current Trends in Management

Eradication of the tubercle bacilli along with prevention of sequel should be primary goal of treatment of EBTB. The treatment of EBTB is similar to pulmonary tuberculosis. Five standard first line drugs are used for the treatment of EBTB: Isoniazid (INH), Rifampin (RIF), Ethambutol (EMB), Pyrazinamide (PZA), and Streptomycin (STR). A six-month regimen consisting of INH, RIF, and PZA for the first two months, followed by INH and RIF for the next 4 months, is the standard treatment. In drug resistant cases, treatment must be based on susceptibility results [33]. Role of DOTS (directly observed treatment short course) in EBTB is not extensively studied.

However, it has been reported that bronchial stenosis may develop in spite of effective antitubercular therapy [2]. Once stenosis has developed, it is not possible to reverse by chemotherapy or steroids. Therefore, at this stage, airway patency must be restored by either endobronchial interventions or surgical means [34, 35].

### 8.1. Controversial Utility of Corticosteroids

Corticosteroid has been used as an adjunct therapy but role of corticosteroids in treatment of endobronchial tuberculosis is still controversial. It has been proposed that steroids may be beneficial in preventing bronchial stenosis because of anti-inflammatory properties [36].

In few of the randomized trials, systemic steroid therapy has improved the endobronchial obstruction due to hilar adenopathy in children [37, 38] but it failed to prevent bronchostenosis in adults [39]. This difference might be due to diverse age groups of these studies as primary (childhood) and secondary (adult) tuberculosis are distinctly different relative to lymph node involvement [40]. The beneficial role in children might be contributed by anti-inflammatory response thereby preventing bronchial compression resulting from erosion of lymph nodes into bronchial lumen [41]. In adults, therapeutic effects may be related to stage of the disease. This modality proves to be valuable in early stages by resolution of inflammation and edema but regression of established fibrostenotic lesions is not possible [40]. Hence, early diagnosis with appropriate therapy before development of fibrosis is essential, to prevent bronchostenosis [39].

### 8.2. Interventional Bronchoscopy: Tackling Bronchostenosis

Interventional bronchoscopy is an alternative treatment strategy to surgical resection in the management of stenosis resulting from endobronchial tuberculosis. There are various bronchoscopic techniques to relieve airway stenosis including laser, cryosurgery [42], controlled heat application [43], balloon dilatation [44, 45], and stent insertion [46, 47].

Airway dilation for palliation of symptoms may be accomplished through rigid and flexible bronchoscopy. The rigid bronchoscope itself may provide dilation with the shear mechanics of the rigid scope. A metal bougie dilator provides a similar effect. When this is not possible, balloon expansion may be useful [48]. Balloon dilatation for tracheobronchial stenosis was first described by Cohen et al. [44]. This method is usually straightforward and minimally invasive and can be performed under local anaesthesia. It is particularly appropriate for annular cicatricial stenosis, since the balloon dilates the stenotic bronchus by stretching and expanding radially. However, one must be cautious to avoid excessive inflation which can result in bronchial wall rupture [48]. As discussed by Shitrit et al. [49], fibrotic process with fixed stenosis may be more amenable to successful balloon dilatation than those with active inflammation, calcification, or carcinoma or in whom the surrounding cartilage was destroyed (malacia). Patients who require more than one session of balloon dilatation are usually in need of stenting or ablative procedures.

Airway stenting is also an important strategy for managing tracheobronchial stenosis. Basically, stenting should be performed after balloon dilatation when the patients prove to be smear negative for tuberculosis. Removable stent is preferable to avoid stent-related complications as long term placement is required. Thus, the Dumon stent is particularly suitable for patients with tuberculous tracheobronchial stenosis but requires rigid bronchoscopy [50]. These silicone stents may be tubular, a Y-configuration that covers portions of the trachea and main stem bronchi, an hourglass configuration with wider ends and a narrower center, or may be customized. On the other hand, metal stents are tubular and may be completely covered, partially covered, or uncovered. Both types of stents suffer from complications of retained secretions, colonisation of stent material, migration, stent fractures, and development of granulation tissue in long term use [51]. To avoid complications and to improve quality of life, any patient who has a stent placed requires appropriate follow-up. It is agreed by all experts in the field that the ideal stent still has to be developed. Newer materials, a better understanding of airway biomechanics, and other approaches, such as bioabsorbable stents, will lead to improvements [52].

Ablative techniques are frequently used which include heat and cold therapies. Ablative heat modalities include electrocautery, argon plasma coagulation (APC), and laser therapy. The use of electrical current for tissue heating is called electrocautery or diathermy. These electrons generate heat for tissue coagulation. For argon plasma coagulation (APC) ionised argon gas jet flow is used to conduct electrons allowing a noncontact mode of treatment. This modality is useful in covering larger surface area to obtain homogenous and superficial necrosis and approaching bronchial segments at an acute angle from the major airways as argon gas quite flexibly flows around bends and corners. APC also helps in clearing blood and mucus while performing superficial coagulation. Electrocautery and APC are better in terms of easy handling and cost-effectiveness when compared with laser therapy [51].

Laser resection uses laser energy delivered via rigid or flexible bronchoscopes in order to manage endobronchial lesions. The neodymium: yttrium aluminium garnet (Nd-YAG) equipment is the most widely used for bronchoscopic interventions because it has sufficient power to vaporise tissues and produces an excellent coagulation effect. Laser bronchoscopy can be performed with the help of rigid or flexible instruments and produces a rapid recanalization of the airway along with immediate relief of obstructive symptoms. The main utility of laser bronchoscopy is in obstructive lesions of the trachea, the left and right main bronchi, and the bronchus intermedius. Laser treatment of obstructions of segmental bronchi does improve ventilation significantly. Complications are rare and can be further restricted by following standardized techniques and safety guidelines. Disadvantages of laser bronchoscopy include the requirement for special training and expensive equipment [52].

Recently, role of mitomycin C is being discussed in laryngotracheal stenosis which can be used as an adjunct to radial incisions made with laser or cautery. Pledgets of cotton soaked in mitomycin C are topically applied to the areas of stenosis. This is believed to impede the inflammatory response [53]. However, further research is required before establishing and claiming its efficacy.

Cryosurgery is also a safer approach as there is no risk of bronchial wall perforation or airway fire. Bronchoscopic cryotherapy consists of cold-induced death of cells by repeated cycles of cold application followed by thawing. Nitrous oxide or liquid nitrogen is most commonly used to produce temperatures of −80°C [54]. Marasso et al. reported their experience with 12 cases of posttubercular stenosis [54]. Both contact and spray cryotherapy have been described. Spray cryotherapy uses a 7-French catheter and nitrogen as a base cryogen. Early results with spray cryotherapy are encouraging but further studies are needed to document its efficacy and safety [55]. The demerits of cryotherapy include time consuming procedure, difficulty in haemostasis, and requirement of repeated procedure [7].

Apart from its established role in treating bronchostenosis, intervention bronchoscopy may also be beneficial in initial stage of tumorous EBTB to prevent grave consequences. In a controlled trial conducted in China, argon plasma coagulation accelerated the healing of tumorous EBTB, thereby preventing the development of bronchial stenosis [56].

### 8.3. Role of Surgery

If all these modalities fail, surgery is considered method of choice since surgical approach offers permanent solution to the problem. Surgical removal of a lobe of lung complicated by atelectasis has been attempted for many years. In addition, new surgical methods, such as sleeve resection, carinal resection, and end-to-end anastomosis, are also being performed nowadays [57, 58]. The above mentioned interventional procedures may play an important preoperative role in establishing airway patency. Futuristic surgical approach may be targeted towards autografts, allografts, bioengineered tracheal platforms, and tracheal transplants [48].

### 8.4. Infection Control

Along with other issues discussed already, control of infection transmission remains a major challenge. Undue delay in making diagnosis due to confusing clinical picture along with poor quality medical care can exacerbate transmission. Therefore, in addition to the efforts targeted towards cure of the disease, simultaneous attempts to control the transmission should be the priority of healthcare providers.

## 9. Future Prospects

Considering the relevance of TGF-*β* in pathogenesis of bronchial stenosis, neutralizing antibodies to TGF-*β*1 may prove beneficial in relieving the obstruction [59]. Still, pathogenesis needs to be explored in detail and introduction of newer therapeutic modalities is desired for favourable outcome.

## 10. Conclusions

Diagnosis of endobronchial tuberculosis should be established early and aggressive treatments must be started to favourably change the course of the disease. Therapy should be individualized according to the stage of the disease demonstrated by bronchoscopic examination. Close follow-up and intervention therapy are essential, specifically in tumorous variety to prevent grave consequences. The ideal approach would be the utilization of these various available technologies along with focus on research to maximize the preventive, palliative, and therapeutic effect.
